# Impaired innate and adaptive immunity of accelerated-aged Klotho mice in sepsis

**DOI:** 10.1186/cc10608

**Published:** 2012-03-20

**Authors:** S Inoue, K Suzuki-Utsunomiya, K Suzuki-Utsunomiya, T Sato, T Chiba, K Hozumi

**Affiliations:** 1 Tokai University, Kanagawa, Japan

## Introduction

Sepsis is primarily a disease of the aged and 60% of sepsis occurs in patients older than 65 years, 80% of deaths due to sepsis occur in this age group. Klotho knockout mice (Klotho mice) develop a syndrome resembling human aging, and exhibit shortened life spans (8 weeks); however, details regarding the immunity of and immunological changes in Klotho mice after sepsis are still unclear. The purpose of the study is to elucidate the immunological changes that occur in Klotho mice after sepsis in order to identify therapeutic targets for sepsis that occurs in aged individuals.

## Methods

(1) Survival study: cecum ligation puncture (CLP) was performed to Klotho and wild-type (WT) mice and 4-day survivals were compared. (2) Cell analysis study: mice were sacrificed at 8 hours post CLP or sham surgery. Spleens, thymus, and serum were harvested for FACS analysis using caspase 3 as a marker for apoptosis, and blood for serum cytokine assay. Bacterial colony count in peritoneal lavage was also analyzed.

## Results

(1) Klotho septic mice started to die from 8 to 12 hours after CLP, and final survival of Klotho mice with CLP was significantly lower than that of WT with CLP (0% vs. 100%, *P *< 0.01). (2) Increased bacterial count in peritoneal cavity and decreased recruitment of neutrophils and macrophages to the peripheral cavity were observed in Klotho-CLP mice. Serum concentration of IL-6, TNF, and IL-10 were significantly higher in Klotho-CLP mice than those in the WT-CLP mice. A dramatically increased caspase 3 positive proportion in Klotho-CLP mice was observed in both flow cytometric and immunohistological analysis (*P *< 0.01).

## Conclusion

Poor survival in Klotho-septic mice may be associated with impaired bacterial clearance with decreased recruitment of neutrophils/macrophages in peritoneal cavity, elevated cytokines in serum, and increased apoptosis in thymus and spleen, following to impaired innate and adaptive immunity.

